# Health risk implications of iron in wastewater soil-food crops grown in the vicinity of peri urban areas of the District Sargodha

**DOI:** 10.1371/journal.pone.0275497

**Published:** 2022-11-08

**Authors:** Shahzad Akhtar, Muhammad Luqman, Muhammad Umer Farooq Awan, Iram Saba, Zafar Iqbal Khan, Kafeel Ahmad, Ahmed Muneeb, Muhammad Nadeem, Aima Iram Batool, Mahpara Shahzadi, Hafsa Memona, Hazoor Ahmad Shad, Ghulam Mustafa, Rana Muhammad Zubair

**Affiliations:** 1 Department of Botany, University of Sargodha, Punjab, Pakistan; 2 Department of Environmental Sciences, University of Veterinary and Animal Sciences (UVAS), Lahore, Pakistan; 3 Department of Botany, GC University Lahore, Lahore, Pakistan; 4 Department of Chemistry, Government College Women University Sialkot, Sialkot, Pakistan; 5 Division of Science and Technology, Department of Botany, University of Education, Lahore, Pakistan; 6 Institute of Food Science and Nutrition, University of Sargodha, Sargodha, Pakistan; 7 Department of Zoology, University of Sargodha, Sargodha, Pakistan; 8 Department of Plant Breeding and Genetics, Ghazi University, Dear Ghazi Khan, Pakistan; 9 Department of Zoology, Queen Mary College, Lahore, Pakistan; 10 Department of Chemistry, Government Associate College Gojra Road Jhang, Punjab, Pakistan; Beijing University of Technology, CHINA

## Abstract

Irrigation using sewage water can be beneficial, as it can increase the productivity of crops but has negative consequences on crops, soil contamination, and human health. It contains a variety of toxins, such as chemicals and heavy metals, which damage the soil and crops. In this regard, the aim of the research was to assess the potential health hazards of iron (Fe) metal in food crops (leafy and root crops) treated with wastewater (T_1), canal water (T_2), and tube well water (T_3). Water, soil, and edible components of food crops were collected at random from three distinct locations. Fe concentration in samples was estimated using atomic absorption spectrophotometer, following wet digestion method. The Fe concentrations, ranged from 0.408 to 1.03 mg/l in water, 31.55 to 187.47 mgkg^-1^ in soil and 4.09 to 32.583 mgkg^-1^ in crop samples; which were within permissible limits of the World Health Organization (WHO). There was a positive correlation between soils and crops. The bioconcentration factor, enrichment factor (EF), daily intake of metals (DIM), health risk index (HRI), and target hazard quotient (THQ) all values were <1, except for a pollution load index >1, which indicated soil contamination, but there was no Fe toxicity in crops, no health risk, and no-carcinogenic risk for these food crops in humans. To prevent the excessive accumulation of Fe metal in the food chain, regular monitoring is needed.

## Introduction

Farmers prefer wastewater over freshwater, even though freshwater is available in some regions because wastewater crops provide a high yield and are hence wastewater is the most beneficial because it contain most beneficial nutrients elements required for growth of crops [1, 2] Because of their easy availability, municipal wastewaters are progressively being utilized as useful resources for irrigation in urban and pre urban agriculture, which may be helpful in alleviating the effluent disposal problem [3]. In terms of availability and nutrient supply, wastewater may be a more reliable source of water than rainfall or groundwater from irrigation systems [4]. Sewage water (SW) contains metals such as Cd, Fe, Cu, Pb, and Zn, which are likely to be introduced into soil and water through a variety of sources, including irrigated water [5]. Metals in polluted soil can pollute crops via root accumulation, and metal content in soil and crops have specific correlations [6]. This might be transmitted from severely polluted agricultural soil to various crop tissues. Metal such as Fe accumulates in food crops, resulting in both positive and hazardous effects on the food chain [7].

Iron is the fourth most prevalent element on the planet [8]. Iron, an important element is essential structural and functional part of every living organism. Iron is an essential nutrient for almost all living things [8–10]. Fe can be found in divalent (Fe^2+^) or trivalent (Fe^3+^) forms. Due to the development of insoluble oxides or hydroxides, Fe^3+^ is not readily utilized by plants or microorganisms [11, 12]. Iron can be taken up by the cortex tissues of roots through the soil, and the reduced Fe^2+^ can enter the xylem and phloem via the Casparian strip [13]. However, a high concentration of Fe^2+^ enters the conducting tissue via the apoplast pathway [14]. Large amount of Fe^2+^ inside the cell produces reactive oxygen species such as superoxide, hydroxyl radicals, and hydrogen peroxide, causing cellular damage [15].

Iron aids in the transportation of oxygen throughout the plant’s roots, leaves, and other organs, resulting in a green colour that indicates a healthy plant [16]. Iron is also required by many plants to perform enzyme processes that keep them alive [17–19]. Fe is essential for maintaining chloroplast structure and function and is involved in the synthesis of chlorophyll in plants [10, 20, 21]. As a component of several key enzymes, including the electron transport chain’s cytochromes, it is required for a wide range of biological functions [8, 9]. Iron is involved in the control of many important functions in plants, including mitochondrial respiration, nucleotide biosynthesis, photosynthesis, nitrogen absorption, hormone regulation, and nutrient transport [22].

The plant uses Fe during its early stages of development. Fe is a vital catalyst in the synthesis of chlorophyll and is also required in many metabolic activities in plants [23]. The lack of Fe can be seen in the light tone of older leaves and the wider veins [24].

Despite the fact, small levels of iron are required for human health. Heme prosthetic groups are found in iron-containing enzymes and proteins and assist in biological oxidation and movement [25]. Fe is also required for the animal body’s blood and physiological functions [15]. Fe deficiency is believed to be present in approximately one-third of the soil [26].

Excessive quantities of Fe in the water results in an overload that might result in diabetes, hemochromatosis, stomach issues, and nausea. The liver, pancreas, and heart can all be harmed by it [7]. Tea with iron has a dark appearance and a bitter, unpleasant flavour [27]. Cooked vegetables in iron-contaminated water become black and unappealing. According to the Environmental Protection Agency’s (EPA) rules for common water sources, iron is designated a secondary pollutant [28]. Secondary standards are used to classify compounds in water that have a nasty taste, froth, colour, aroma, corrosion, or pigment and yet have no obvious health impacts [29].

Farmers are forced to use wastewater for irrigation due to the harsh conditions caused by water scarcity. Aside from its beneficial purpose, it exposes people to heavy metals by using wastewater-irrigated food crops. Numerous investigations on the toxicity of the heavy metals Zn, Cr, Fe, Mn, and Cu in wastewater-irrigated fodders have been conducted in various parts of Pakistan [30–33].

The current study was conducted to (1) check the effect of wastewater irrigation on Fe uptake by food crops, (2) evaluate the transfer of Fe from soil to food crops, (3) identify the pollution incidence of soil due to Fe, and (4) assess the health hazard to consumers through the intake of Fe-contaminated food plants.

## Materials and methods

**Fig 1:**
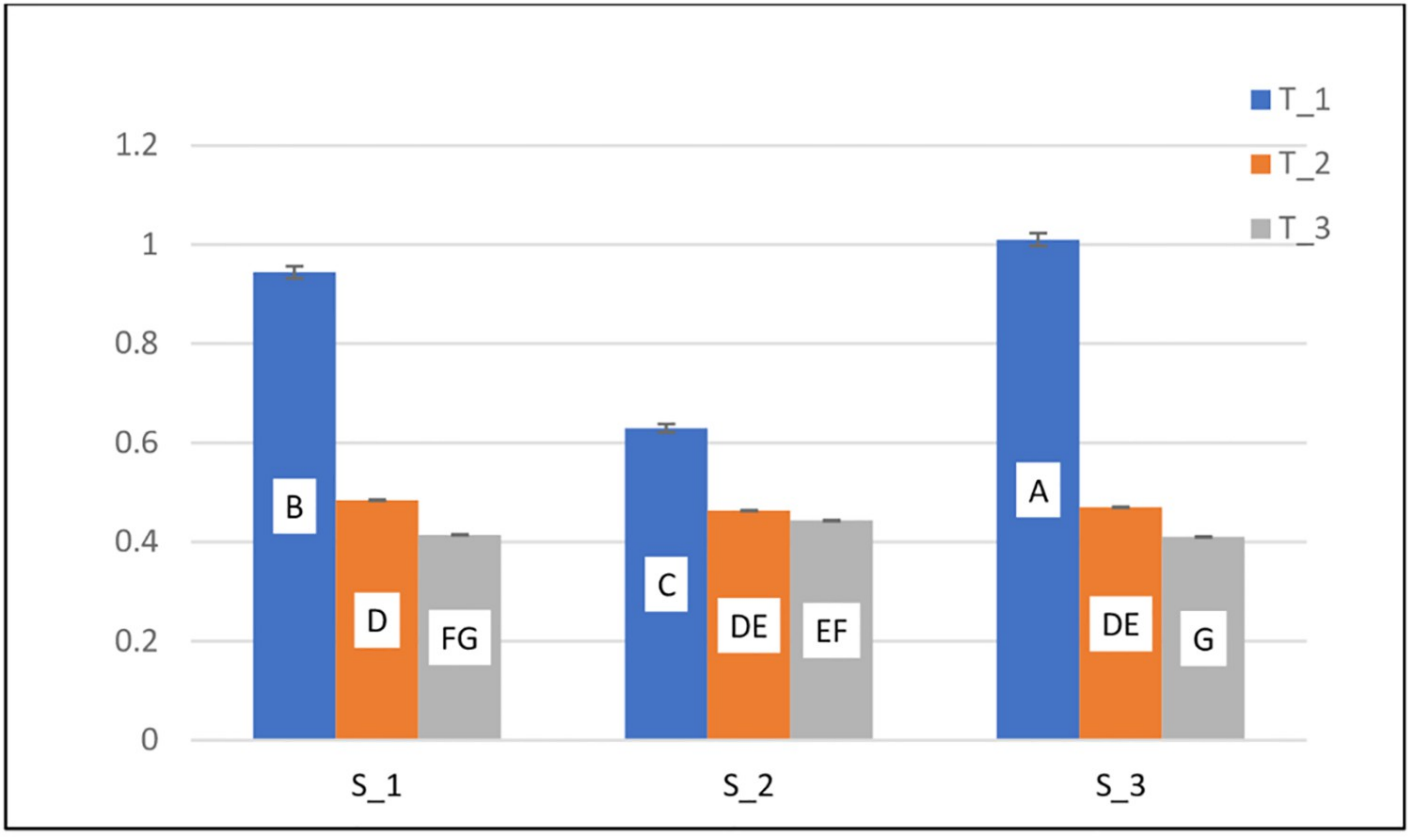
Study map.

The current study was carried out in three Tehsils (Sargodha, Sahiwal, and Shahpur) of the District Sargodha in the Punjab province of Pakistan. This study was conducted in former field that was divided into three sites, each situated in a distinct location in three tehsils of the Sargodha district and treated with diverse irrigated sources, namely, wastewater (T_1) (sewage and domestic house waste water), canal water (T_2), and tube-well water (T_3). The soil and crop samples (leafy and root vegetables) were obtained from 3 different places in the district of Sargodha (Fig 1). The coordinates of sites were 32°03’39.7"N 72°37’58.1"E, 31°53’37.5"N 72°25’21.0"E, and 32°17’30.1"N 72°25’01.8"E for S_1, S_2, and S_3 respectively. To collect samples, the Randomized Complete Block Design (RCBD) was used. Each site (S_1, S_2, and S_3) had eleven food crops (7 leafy and 4 root vegetables) with three treatments (T_1, T_2, and T_3) and three replicates of each crop sample (11 x 3 x 3 x 3). During the years 2018–2019, food crop specimens were taken.

The sampling of soil was done by using the Rhue and Kidder [34] method, and vegetable sampling was done by using the techniques of Akhtar, Khan [35].

Food crop edible components were collected at random from fields treated with wastewater, tube well water, and canal water (Table 1). Soil and food crop samples were digested using the wet digestion method. An atomic absorption spectrophotometer (Shimadzu Co., Ltd., Japan) was used to measure the quantity of Fe in the soil and food crops. Prior to usage, the device was calibrated. The analysis was performed using the Standard Reference Material (SRM 1570) for Fe metal from the National Institute of Standard Technology to assure precision and accuracy. Acetylene was flowing at a rate of 2.2 L/min, with a wavelength of 248. 3 nm, a slit width of 0.2 nm, a lamp current of 12 mA, and a burner height of 9 mm. Every sample had its metal content verified three times. All outcomes were in accordance with international standards [35]. The following quality standards were applied to ensure that the study’s findings were acceptable. Sigma Aldrich, Merck (Germany), and BDH provided the analytical-grade chemicals for the experiment (UK). In each sample, metals were detected in triplicate. The results were entirely in accordance with international standards.

**Table 1:** List of selected sampled food crops and their parts used for analysis.

Sr #	English Name	Scientific Names	Parts used for analysis
**1**	**Spinach**	***Spinacia oleracea***	**Leaves**
**2**	**Mustard**	***Brassica campestris***	**Leaves**
**3**	**Coriander**	***Coriandrum sativum***	**Leaves/**
**4**	**Mint**	***Mentha spicata***	**Leaves**
**5**	**Fenugreek**	***Trigonella foenum-graecum***	**Leaves**
**6**	**Lettuce**	***Lactuca sativa***	**Leaves**
**7**	**Chenopodium**	***Chenopodium album***	**Leaves**
**8**	**Carrot**	***Daucus carota***	**Tuber**
**9**	**Radish**	***Raphonus sativus***	**Tuber**
**10**	**Beetroot**	***Beta vulgaris***	**Tuber**
**11**	**Turnip**	***Brassica rapa***	**Tuber**

### Pollution indices

#### Bioconcentration factor (BCF)

The BCF was calculated by Eq 1 [36].


$$BCF=\frac{\left(M\right)\;Food\;Crop}{\left(M\right)\;Soil}$$ (1)

The M indicates the metal concentration (mgkg^-1^). A BCF value greater than one implies that hazardous metals were found in high concentrations in food crops. BCF values of 0.01 were found in non-accumulator plants, 0.1–1 in moderate accumulators, and 1–10 in hyperaccumulator plants (Netty et al., 2013).

#### Pollution load index (PLI)

The pollution load index was calculated by Eq 2 [35].

$$PLI=\frac{{\left(M\right)}^{IS}}{{\left(M\right)}^{RS}}$$ (2)

where M is the metal content (mgkg^-1^), IS is the metal (mgkg^-1^) in the investigated soil where crops were grown, RS is the reference value of the metal in the soil, PLI 1 indicates that there was no heavy metal contamination, and PLI > 1 or equal to 1 indicates that there was a high level of pollutants and poor soil condition [35].

#### Enrichment factor (EF)

Using the formula Buat-Menard and Chesselet [37], the enrichment factor was calculated by Eq 3.


$$\text{EF}=\frac{\text{Metal}\left(\frac{\text{Food}\;\text{crop}}{\text{Soil}}\right)\text{Sample}}{\text{Metal}\left(\frac{\text{Food}\;\text{crop}}{\text{Soil}}\right)\text{Refrence}\;\text{value}}$$ (3)

[38, 39] proposed reference levels for metals in soil (56.9 mgkg^-1^) and food crop (425.5 mgkg^-1^) samples, respectively.

If the enrichment factor (EF) is <1, it means there was no enrichment, 1–2 means there was modest enrichment, 3–4 means moderate enrichment, 5–9 means moderate-severe enrichment, 10–24 means severe enrichment, 25–49 means extremely severe enrichment, and 50 or higher means extremely severe enrichment [35].

#### Daily Intake of Metals (DIM)

The daily intake of heavy metals is determined by the metal concentration in crops and the daily intake of the examined food crop by Eq 4 [40].

$$\text{DIM}=\;\frac{\text{C}\;\left(\text{Metal}\right)\times \text{D}\;\left(\text{Food}\;\text{intake}\right)}{\text{B}\;\left(\text{average}\;\text{weight}\right)}$$ (4)

where D (food intake) is the daily intake of a food crop (mgkg^-1^) 0.345 (kg/person), C (metal) is the heavy metal concentration in the food crop (mgkg^-1^), and B is the average body weight (65 kg), calculated by taking the average weight of 100 adult males/females during research work at the University of Sargodha.

#### Health Risk Index (HRI)

DIM was divided by the reference oral dose as given in Eq 5 to obtain the health risk index (HRI).

$$\text{HRI}=\frac{\text{DIM}}{\text{RfD}}$$ (5)

where DIM is the daily intake metal and RfD is the oral reference dose for metals (Table 3). To quantify the dangers associated with consuming heavy metal-polluted food crops, a health risk score was calculated. People will be safe to consume those sorts of crops if the HRI is less than 1 [36]. According to reports, if the HRI was greater than one, the consumer was at risk [41].

#### Target hazard quotient (THQ)

The THQ identifies the hazardous metal’s non-carcinogenic health risk that is calculated by Eq 6 given by [Chien, Hung [42]].

$$\text{THQ}=\left(\frac{\text{C}\;\text{x}\;\text{DI}\;\text{x}\;\text{EF}\;\text{x}\;\text{ED}\;\text{total}}{\text{RfD}\;\text{x}\;\text{Bw}\;\text{x}\;\text{ATn}}\right)\;\text{X}\;10{\;}^{-3}$$ (6)

where C is the heavy metal concentration in the food crop, DI is the daily intake of 0.345 kg was considered a typical serving for a day of food crop, EF stands for exposure frequency, which was measured in no. of days per year (365 days/year), ED was exposure duration that was 30 years or 10950 days RfD was reference oral dose (mgkg^-1^/day) Bw was average bodyweight that was 65Kg ATn was 60 years or days 21900. If the value of THQ was less than 1, it indicates no significant carcinogenic risk. In contrast, values of THQ were higher than 1, indicating a greater carcinogenic effect.

### Statistical analysis

All results of the samples were subjected to analysis by using the software Microsoft Excel and Minitab 16, and the data from each attribute were statistically analysed. To discover significant differences between mean values, a three-factor factorial design (three-way ANOVA) was used for the analysis of soils and crops. Metal transfer correlation analysis was performed. In addition to ANOVA, every treatment’s mean was compared to the means of other treatments.

## Results

### Iron concentration in water

**Fig 2:**
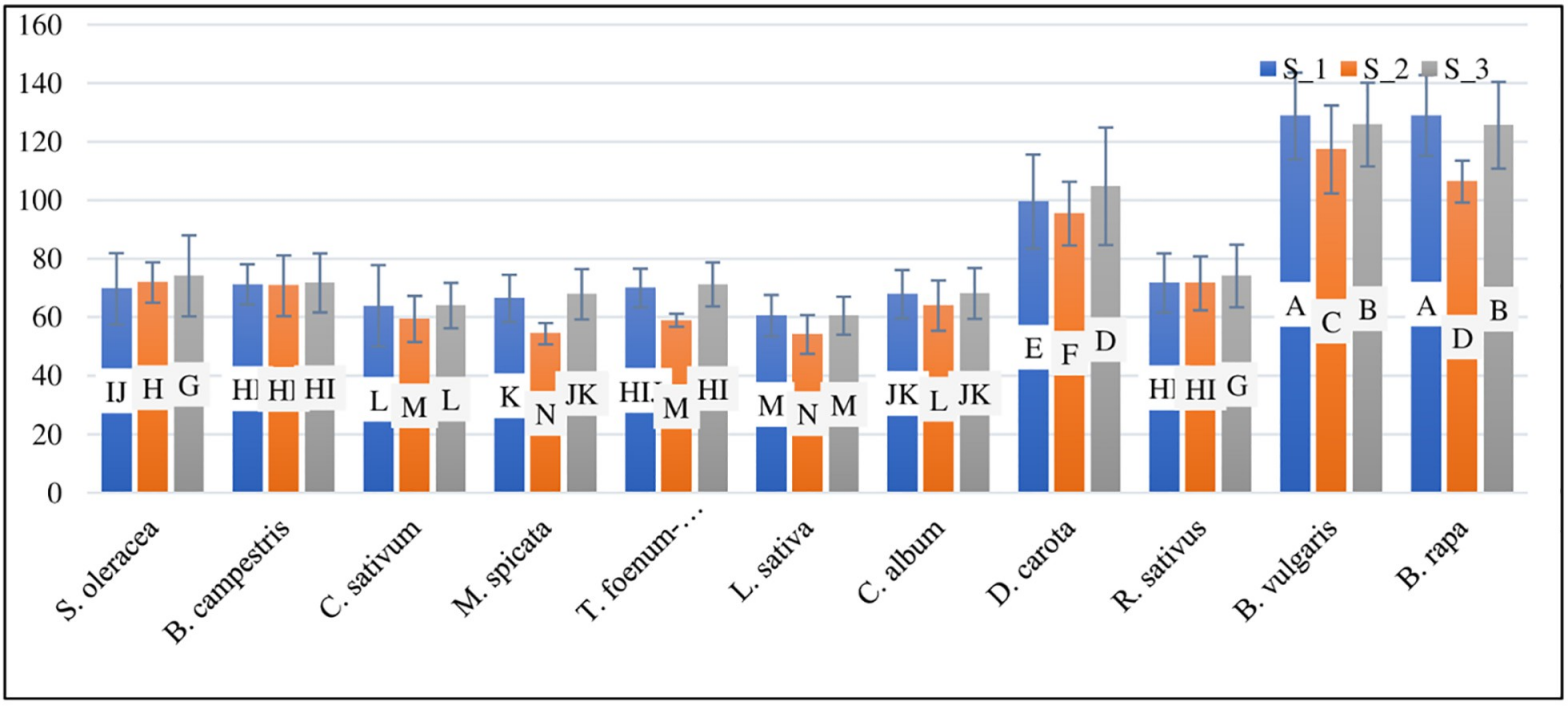
Fe (concentrations in mg/L) in water at three sites (S-1, S-2, and S-3).

**The** Fe concentrations in water varied significantly (P<0.05) by site, treatment, and site x treatment (Table 2). The concentrations of Fe in the samples ranged from 0.408 to 1.03 mg/l. At S_3, the concentration of iron was lower in T_3 and higher in T_1. At all sites, the descending order of iron metal in water was T_1 > T_2 and T_3 (Fig 2).

**Table 2:** Analysis of variance for Fe in water.

Source	DF	Water
**Site**	**2**	0.037*
**Treatment**	**2**	0.519*
**Site x Treatment**	**4**	0.044*
**Error**	**18**	
**Total**	**26**	

where * is significant at the 0.001 level.

### Iron concentration in soils

**Fig 3:**
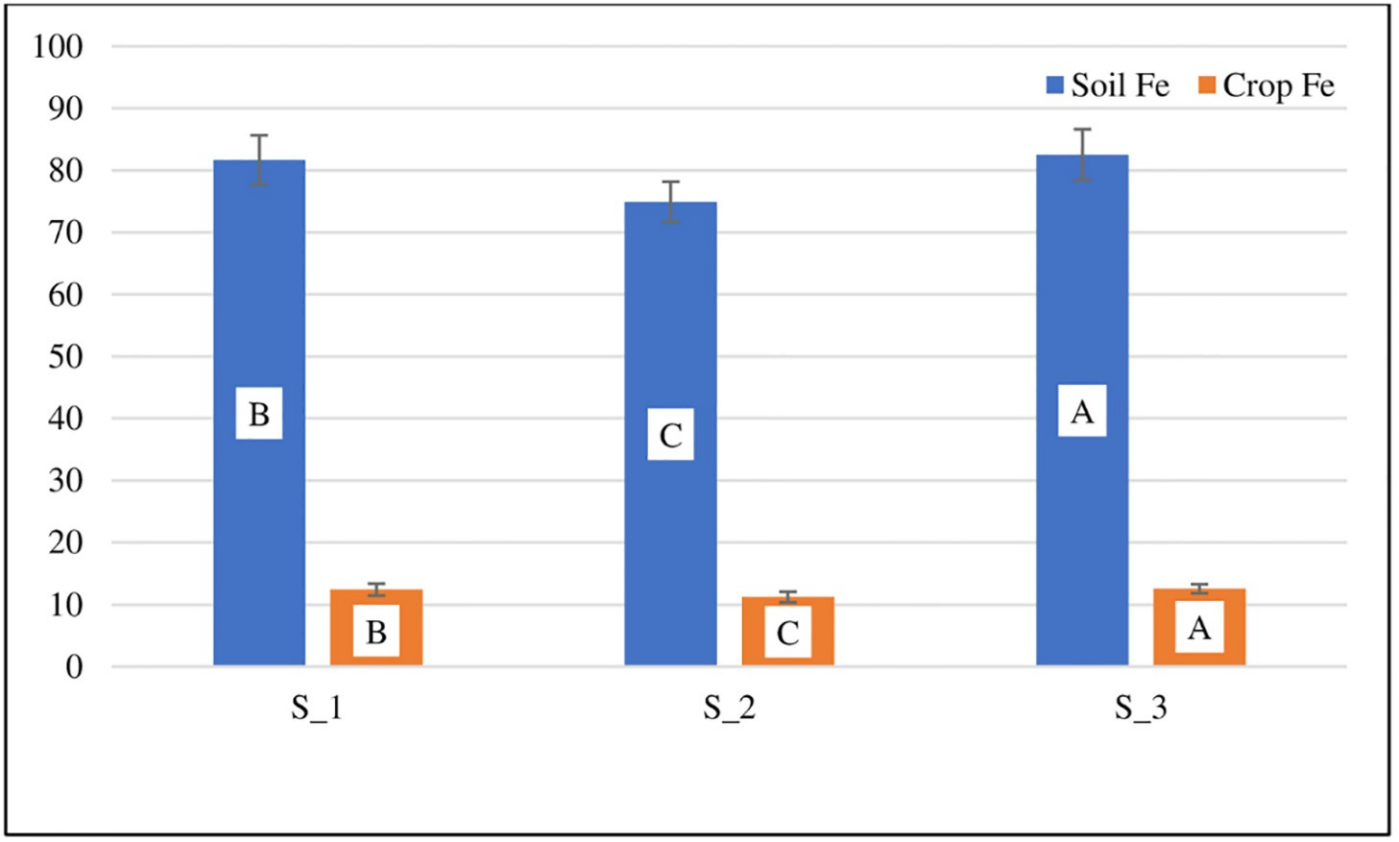
Fe (Fe concentrations in mgkg^-1^) in soil at S_1, S_2 & S_3.

**Fig 4:**
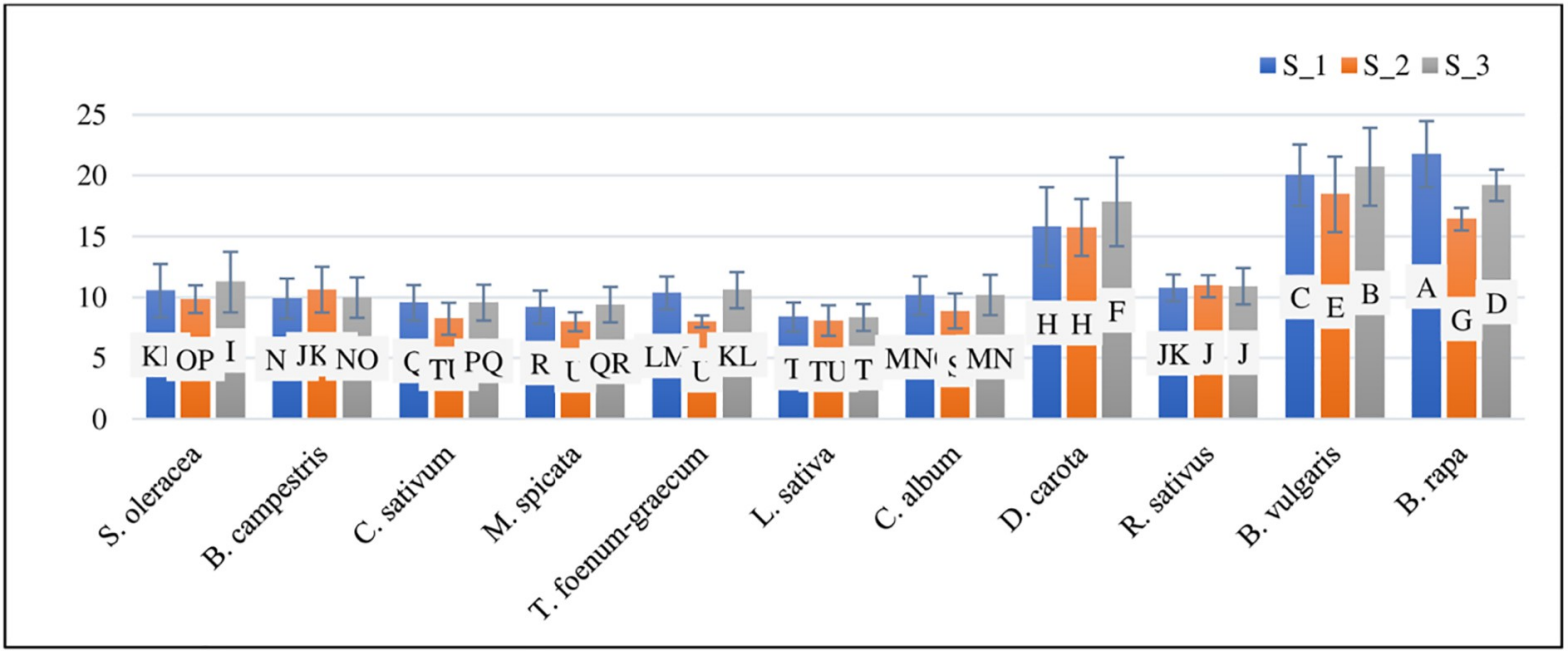
(Fe concentrations in mgkg^-1^) at different sites in soils and crops.

The analysis of variance showed that the iron concentration in the soil at different sites treated with different water sources showed highly significant results (Table 3). The mean iron concentrations were 116.58, 71.91, and 50.64 mgkg^-1^ in the T_1, T_2, and T_3 treatments, respectively. The Fe concentration ranged from a minimum of 31.55 mgkg^-1^ to a maximum of 187.47 mgkg^-1^ at T_3 and T_1, respectively (Table 4). The descending order of Fe metal in soil at S_1 was *B*. *rapa* > *B*. *vulgaris* > *D*. *carota* > *B*. *campestris* > *T*. *foenum-graecum* > *S*. *oleracea* > *C*. *album* > *M*. *spicata* > *C*. *sativum* and *L*. *sativa*. *B*. *vulgaris* > *B*. *rapa* > *D*. *carota* > *S*. *oleracea* > *B*. *campestris* > *C*. *album* > *C*. *sativum* > *T*. *foenum-graecum* > *M*. *spicata* and *L*. *sativa* were found to have the highest levels of Fe metal in soil at S_2. The descending order of Fe metal in soil at S_3) was reported to be *B*. *vulgaris* > *B*. *rapa* > *D*. *carota* > *S*. *oleracea* > *B*. *campestris* > *T*. *foenum-graecum* > *C*. *album* > *M*. *spicata* > *C*. *sativum* and *L*. *sativa* (Fig 3). At S_1, S_2, and S_3, the mean values of soil Fe were 81.26, 74.92, and 82.52, respectively (Fig 4).

**Table 3:** Analysis of variance for Fe in soils and crops.

Source	DF	Mean Squares (Soils)	Source	DF	Mean Squares (Crops)
**Site**	2	1722*	Site	2	53.77*
**Treatments**	2	112132*	Treatments	2	3343.49*
**Soils**	10	15065*	Crops	10	486.76*
**Site x Treatments**	4	1888*	Sites x Treatments	4	60.11*
**Sites x Soils**	20	132*	Sites x Crops	20	7.54*
**Sites x Treatments**	20	1179*	Sites x Crops	20	53.49*
**Sites x Treatments x Soils**	40	240*	Sites x Treatments x Crops	40	11.45*
**Error**	198	1	Error	198	0.03
**Total**	296		Total	296	

Where * is significant at the 0.001 level.

**Table 4:** Fe concentration in soil and crops with T_1, T_2 & T_3.

Variable	Treatment	Mean	SE Mean	Minimum	Maximum
**Soil Fe**	T_1	116.58	2.57	64.32	187.47
	T_2	71.91	2.24	47.99	118.13
	T_3	50.64	1.62	31.55	91.88
**Crop Fe**	T_1	18.432	0.663	9.697	32.583
	T_2	10.63	0.429	6.737	19.764
	T_3	7.07	0.295	4.09	14.908

### Iron concentration in crops

ANOVA revealed very significant results for Fe accumulation in edible portions of food crops growing at three sites (S_1, S_2, and S_3) treated with T_1, T_2, and T_3 sources of water (Table 3). The mean iron concentration in crops was 18.43 mgkg^-1^ at T_1, 10.63 mgkg^-1^ at T_2, and 7.07 mgkg^-1^ at T_3. The Fe content ranged from 4.09 mgkg^-1^ to 32.583 mgkg^-1^ in the T_3 and T_1 treatment, respectively (Table 4). At S_1, S_2, and S_3, the mean values of crop Fe were 12.3, 11.197, and 12.53, respectively (Fig 4).

**Fig 5:**
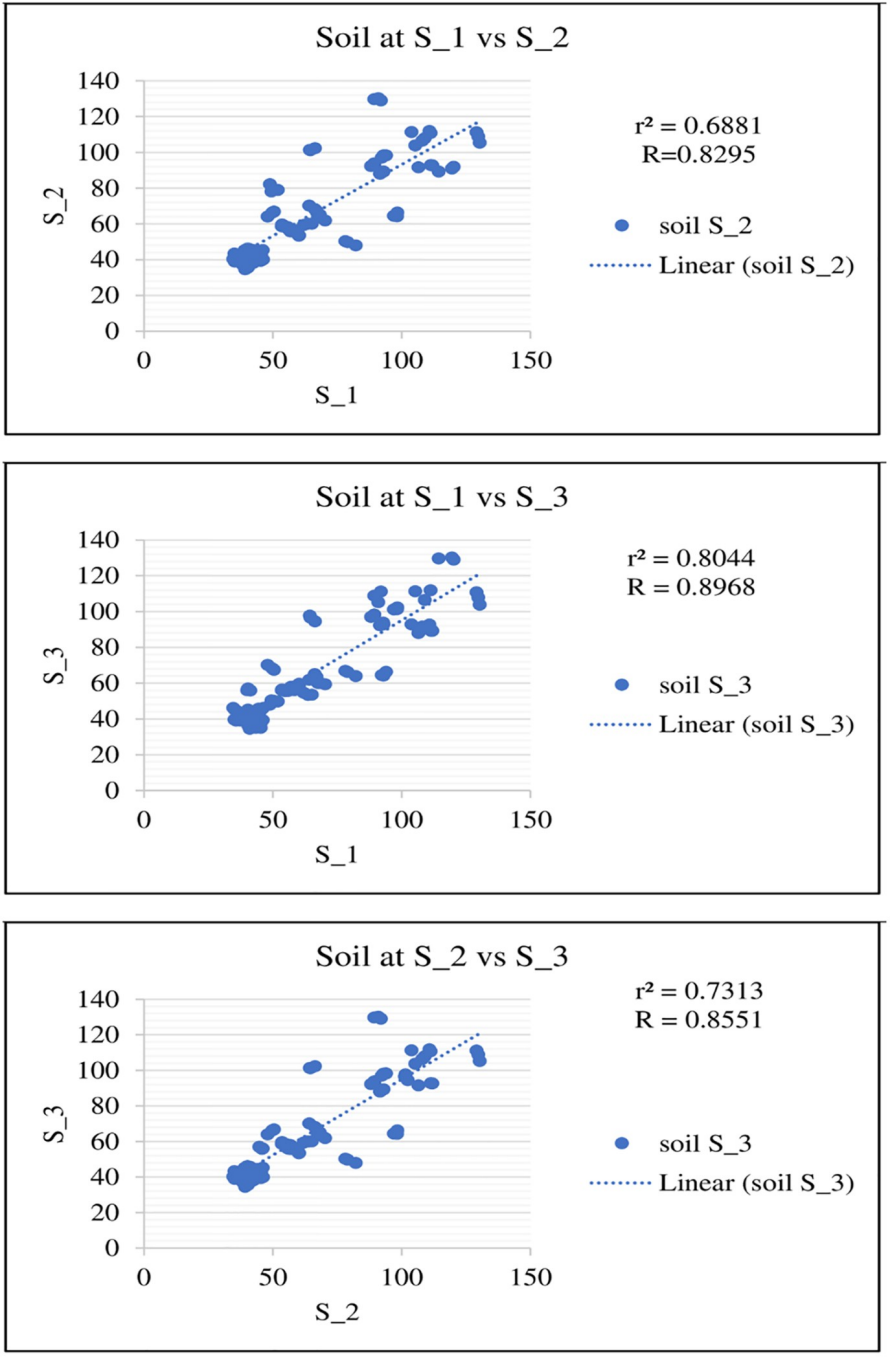
Scatter plot for Fe concentration in soil at S_1, S_2, and S_3.

The descending order of Fe metal in crops at S_1 was *B*. *rapa* > *B*. *vulgaris* > *D*. *carota* > *R*. *sativus* > *S*. *oleracea* > *T*. *foenum-graecum* > *C*. *album* > *B*. *campestris* > C. sativum > M. *spicata and L*. *sativa*; at S_2, it was *B*. *vulgaris* > *B*. *rapa* > *D*. *carota* > *R*. *sativus* > *B*. *campestris* > *S*. *oleracea* > *C*. *album* > *C*. *sativum* > *L*. *sativa* > *T*. *foenum-graecum* and *M*. *spicata*; and at S_3, it was *B*. *vulgaris* > *B*. *rapa* > *D*. *carota* > *S*. *oleracea* > *R*. *sativus* > *T*. *foenum-graecum* > *C*. *album* > *B*. *campestris* > *C*. *sativum* > *M*. *spicata* and *L*. *sativa* (Fig 5). The Fe concentration was higher in T_1-treated crops because the iron concentration in irrigated water was higher than that built up in soil and transferred to the crops.

### Scatter plot analysis for Fe concentration in soils and crops

**Fig 6:**
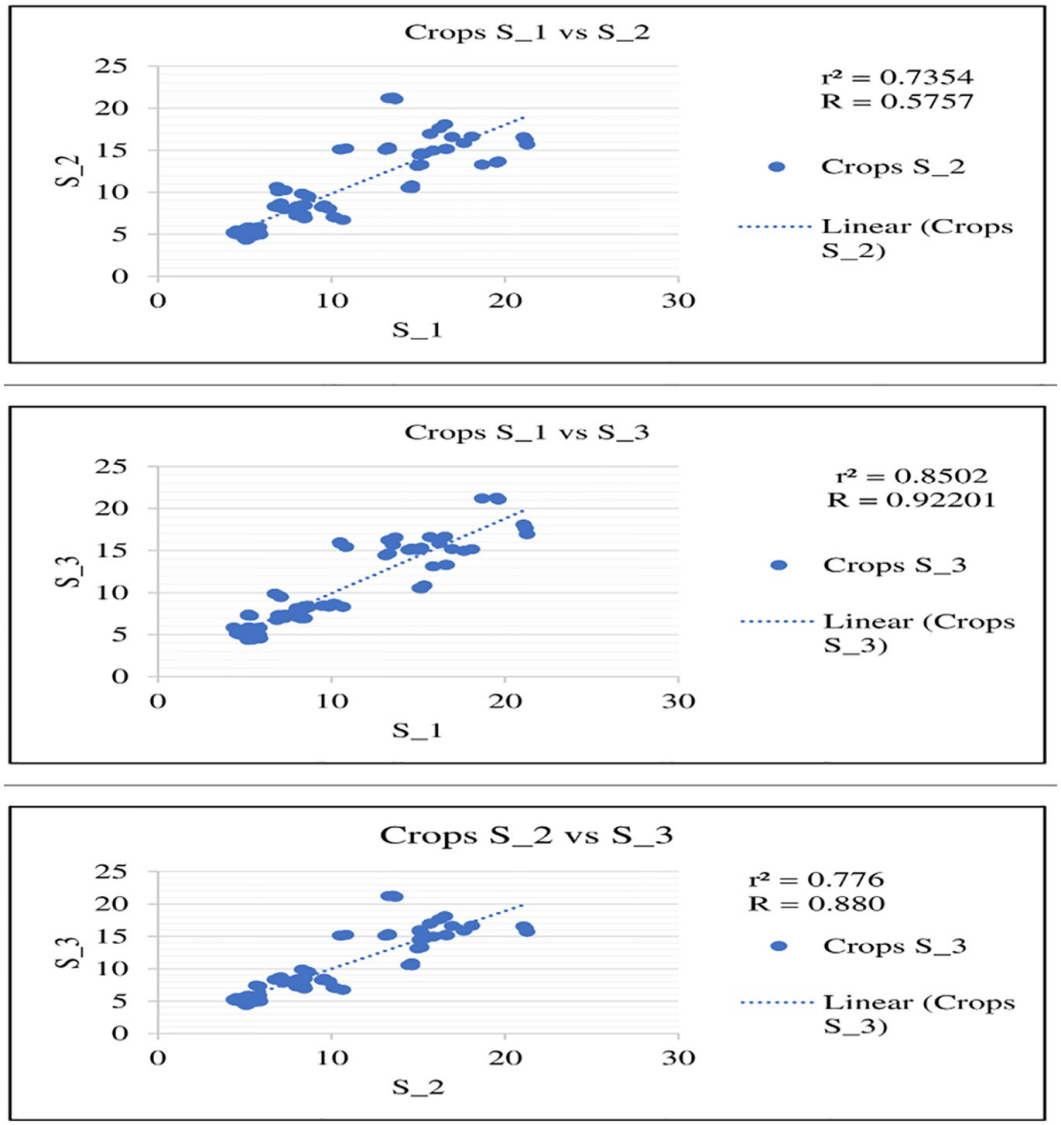
Scatter plot for Fe concentration in crops at S_1 vs S_2, S_1 vs S_3, and S_2 vs S_3.

**Fig 7:**
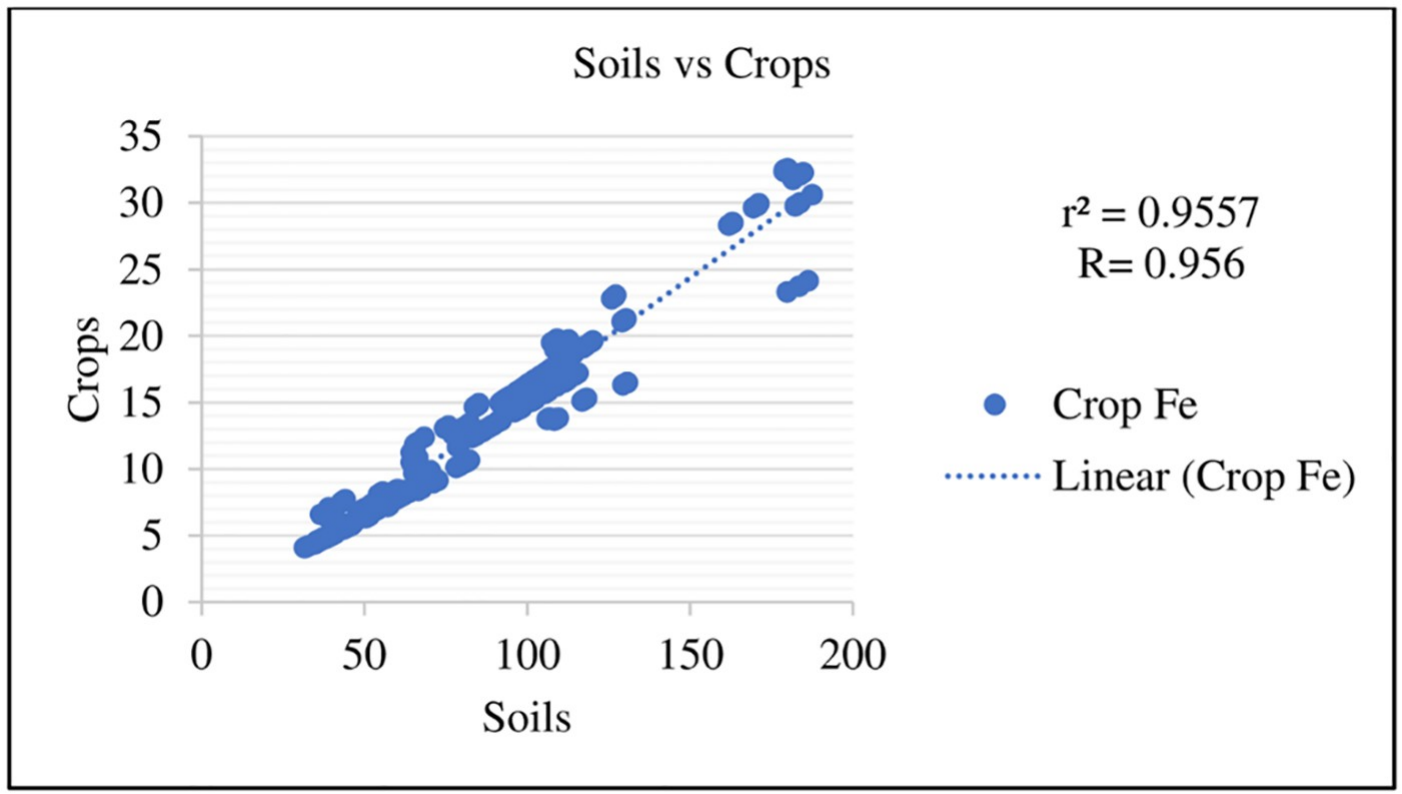
Scatter plot of soil and crop correlations.

Scatter plots were generated to compare the Fe concentrations in soils and crops at each site, S_1 vs S_2, S_1 vs S_3, and Site -2 vs S_3, which showed a high positive correlation at the site level (Figs 5 and 6). Similarly, a very high positive correlation in soils vs crops was also observed (Fig 7). The positive correlation shows the synergistic effect of Fe on all sites.

### Bioconcentration factor, pollution load index and enrichment factor of iron

**Fig 8:**
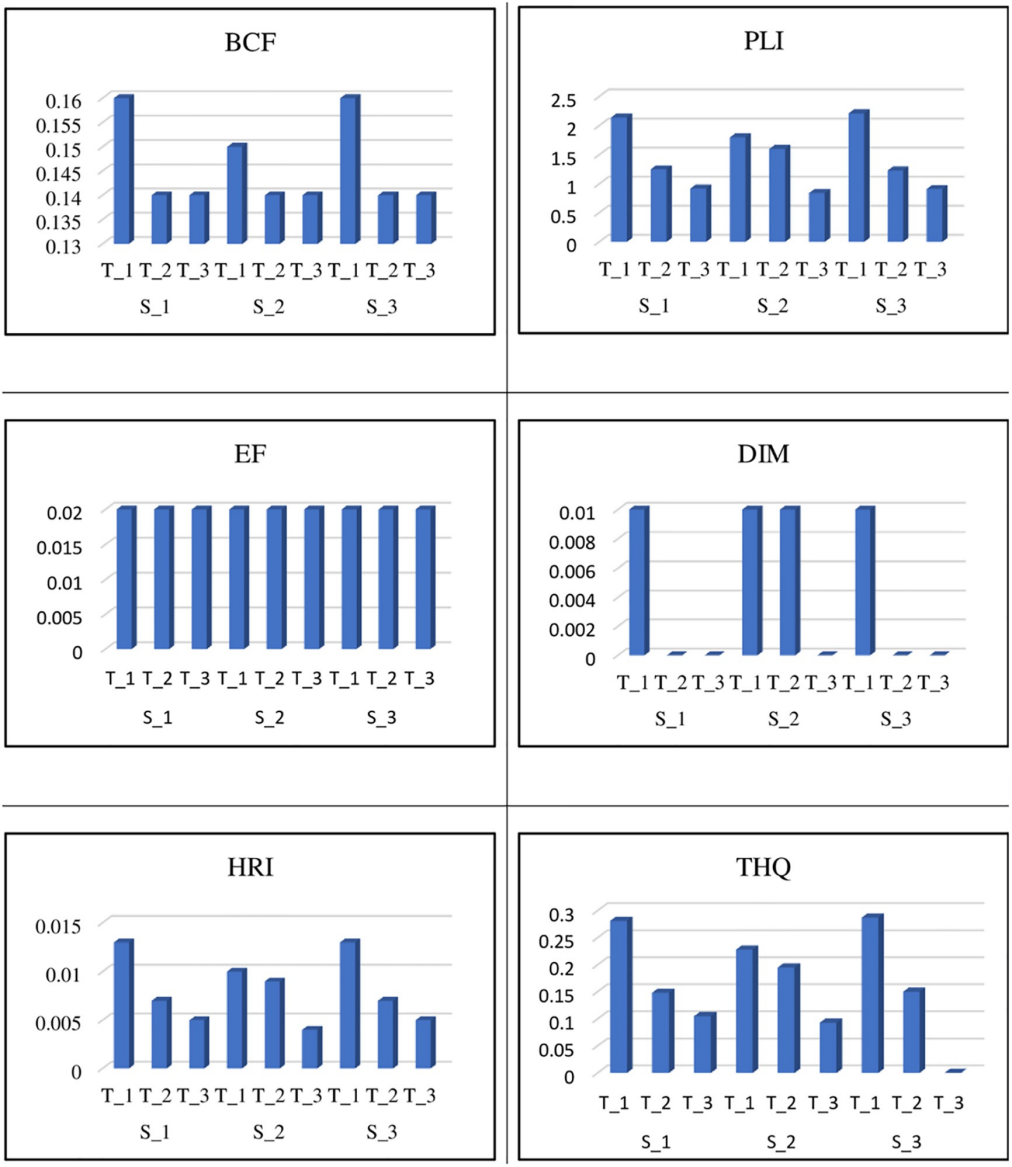
Mean values of bioconcentration factor (BCF), pollution load index (PLI), enrichment factor (EF), daily intake of metals (DIM), health risk index (HRI), and target hazard quotient (THQ) at three sites (S_1, S_2, and S_3) and treatments (T_1, T_2, and T_3).

The Fe bioconcentration factor ranged from 0.12 to 0.19. *C*. *sativum* had the lowest BCF at S_3, while *B*. *vulgaris* had the highest BCF at S_3 when irrigated with T_3 and T_1 (Table 5). The mean value of the bioconcentration factor was found to be maximum at T_1 treated S_1 and S_3 (Fig 8).

**Table 5:** Bioconcentration factor (BCF), pollution load index (PLI), enrichment factor (EF), daily intake of metals (DIM), health risk index (HRI), and target hazard quotient (THQ) values at three sites (S_1, S_2, and S_3) and treatments (T_1, T_2, and T_3).

Crops	BCF								
	S_1			S_2			S_3		
	T_1	T_2	T_3	T_1	T_2	T_3	T_1	T_2	T_3
***S*. *oleracea***	0.16	0.14	0.13	0.15	0.13	0.13	0.16	0.14	0.13
***B*. *campestris***	0.15	0.13	0.13	0.16	0.14	0.13	0.15	0.13	0.13
***C*. *sativum***	0.16	0.14	0.13	0.15	0.13	0.13	0.16	0.14	0.12
***M*. *spicata***	0.15	0.13	0.13	0.16	0.14	0.13	0.15	0.13	0.13
***T*. *foenum-graecum***	0.16	0.14	0.13	0.15	0.13	0.13	0.16	0.14	0.13
***L*. *sativa***	0.15	0.13	0.13	0.16	0.14	0.13	0.15	0.13	0.13
***C*. *album***	0.16	0.14	0.13	0.15	0.15	0.13	0.16	0.14	0.13
***D*. *carota***	0.17	0.15	0.13	0.18	0.18	0.13	0.17	0.17	0.15
***R*. *sativus***	0.13	0.18	0.16	0.13	0.13	0.17	0.15	0.13	0.18
***B*. *vulgaris***	0.16	0.13	0.17	0.17	0.17	0.13	0.19	0.16	0.13
***B*. *rapa***	0.17	0.17	0.15	0.13	0.13	0.16	0.13	0.17	0.17
**PLI**									
***S*. *oleracea***	2.07	0.88	0.72	1.6	1.4	0.79	2.28	0.87	0.76
***B*. *campestris***	1.91	1.16	0.69	1.89	1.21	0.63	1.93	1.12	0.73
***C*. *sativum***	1.62	1.05	0.69	1.56	0.95	0.62	1.64	1.04	0.7
***M*. *spicata***	1.72	0.99	0.8	1.14	1.01	0.71	1.79	0.99	0.8
***T*. *foenum-graecum***	1.69	1	0.99	1.16	1.06	0.89	1.78	1.03	0.95
***L*. *sativa***	1.52	0.99	0.67	1.37	0.92	0.56	1.48	1	0.7
***C*. *album***	1.75	1.04	0.79	1.71	1.71	0.7	1.8	0.96	0.83
***D*. *carota***	2.86	1.38	1.01	2.23	2.23	0.94	3.24	1.32	0.97
***R*. *sativus***	1.91	1.17	0.7	1.87	1.87	0.76	1.97	1.26	0.67
***B*. *vulgaris***	3.24	2.06	1.48	2.99	2.99	1.18	3.15	2.06	1.42
***B*. *rapa***	3.22	1.98	1.61	2.29	2.29	1.42	3.22	1.91	1.49
**EF**									
***S*. *oleracea***	0.022	0.019	0.017	0.02	0.017	0.017	0.022	0.019	0.017
***B*. *campestris***	0.02	0.017	0.017	0.022	0.019	0.017	0.02	0.017	0.017
***C*. *sativum***	0.022	0.019	0.017	0.02	0.017	0.017	0.022	0.019	0.017
***M*. *spicata***	0.02	0.017	0.017	0.022	0.019	0.017	0.02	0.017	0.017
***T*. *foenum-graecum***	0.022	0.019	0.017	0.02	0.017	0.017	0.022	0.019	0.017
***L*. *sativa***	0.02	0.017	0.017	0.022	0.019	0.017	0.02	0.017	0.017
***C*. *album***	0.022	0.019	0.0164	0.02	0.02	0.017	0.022	0.019	0.017
***D*. *carota***	0.023	0.02	0.017	0.024	0.024	0.017	0.023	0.023	0.02
***R*. *sativus***	0.017	0.024	0.022	0.017	0.017	0.023	0.02	0.017	0.024
***B*. *vulgaris***	0.022	0.017	0.023	0.023	0.023	0.017	0.025	0.022	0.017
***B*. *rapa***	0.023	0.023	0.02	0.017	0.017	0.022	0.017	0.023	0.023
**DIM**									
***S*. *oleracea***	0.0087	0.00317	0.0024	0.0061	0.00467	0.00257	0.00956	0.00313	0.00253
***B*. *campestris***	0.00729	0.00385	0.00224	0.00792	0.00435	0.00211	0.00738	0.00372	0.00237
***C*. *sativum***	0.00681	0.00379	0.0023	0.00597	0.00315	0.00201	0.00686	0.00374	0.00233
***M*. *spicata***	0.00657	0.00328	0.00258	0.00479	0.00364	0.00237	0.00683	0.00329	0.0026
***T*. *foenum-graecum***	0.0071	0.00361	0.0033	0.00442	0.00352	0.0029	0.00745	0.00372	0.00315
***L*. *sativa***	0.00582	0.00331	0.00219	0.00574	0.00332	0.00188	0.00567	0.00334	0.00227
***C*. *album***	0.00735	0.00374	0.00262	0.00652	0.00652	0.00226	0.00756	0.00347	0.00275
***D*. *carota***	0.01284	0.0053	0.00326	0.01035	0.01035	0.00313	0.01454	0.00592	0.0037
***R*. *sativus***	0.00621	0.00545	0.00292	0.00623	0.00623	0.00342	0.00755	0.00409	0.00312
***B*. *vulgaris***	0.01359	0.00687	0.00665	0.01345	0.01345	0.00381	0.01465	0.00865	0.00473
***B*. *rapa***	0.01444	0.00887	0.00614	0.00742	0.00742	0.00595	0.01071	0.0086	0.00669
**HRI**									
***S*. *oleracea***	0.012	0.005	0.003	0.009	0.007	0.004	0.014	0.004	0.004
***B*. *campestris***	0.01	0.005	0.003	0.011	0.006	0.003	0.011	0.005	0.003
***C*. *sativum***	0.01	0.005	0.003	0.009	0.005	0.003	0.01	0.005	0.003
***M*. *spicata***	0.009	0.005	0.004	0.007	0.005	0.002	0.01	0.005	0.004
***T*. *foenum-graecum***	0.01	0.005	0.005	0.006	0.005	0.004	0.011	0.005	0.005
***L*. *sativa***	0.008	0.005	0.003	0.008	0.005	0.003	0.008	0.005	0.003
***C*. *album***	0.011	0.005	0.004	0.009	0.009	0.003	0.011	0.005	0.004
***D*. *carota***	0.018	0.008	0.005	0.015	0.015	0.004	0.021	0.008	0.005
***R*. *sativus***	0.009	0.008	0.004	0.009	0.009	0.005	0.011	0.006	0.004
***B*. *vulgaris***	0.019	0.01	0.009	0.019	0.019	0.005	0.021	0.012	0.007
***B*. *rapa***	0.022	0.013	0.009	0.011	0.011	0.008	0.015	0.012	0.01
**THQ**									
***S*. *oleracea***	0.28	0.1	0.08	0.19	0.15	0.08	0.31	0.1	0.08
***B*. *campestris***	0.23	0.12	0.07	0.25	0.14	0.07	0.24	0.12	0.08
***C*. *sativum***	0.22	0.12	0.07	0.19	0.1	0.055	0.22	0.12	0.07
***M*. *spicata***	0.21	0.1	0.08	0.15	0.12	0.08	0.22	0.1	0.08
***T*. *foenum-graecum***	0.23	0.12	0.11	0.14	0.11	0.09	0.24	0.12	0.1
***L*. *sativa***	0.19	0.11	0.07	0.18	0.11	0.06	0.18	0.11	0.07
***C*. *album***	0.23	0.12	0.08	0.21	0.21	0.07	0.24	0.11	0.09
***D*. *carota***	0.41	0.17	0.1	0.33	0.33	0.1	0.46	0.19	0.12
***R*. *sativus***	0.2	0.17	0.09	0.2	0.2	0.11	0.24	0.13	0.1
***B*. *vulgaris***	0.43	0.22	0.21	0.43	0.43	0.12	0.47	0.28	0.15
***B*. *rapa***	0.46	0.28	0.2	0.24	0.24	0.19	0.34	0.27	0.21

The PLI value of Fe ranged from 0.56 to 3.24. PLI was lowest in *L*. *sativa* at S_2 and greatest in *D*. *carota* at S_3 watered with T_3 and T_1, respectively (Table 5). The mean value of the pollution load index was found to be maximum at T_1-treated Crops at S_3 (Fig 8).

The Fe enrichment factor ranged from 0.0164 to 0.025. In *C*. *album*, the lowest EF was reported at S_1, and the greatest EF was observed at S_3 under irrigation with T_3 and T_1 (Table 5). The mean value of the enrichment factor was found to be similar at all sites and treatments (Fig 8).

### Daily intake of iron health risk index and target hazard quotient of iron

The daily intake of Fe metal ranged from 0.00188 to 0.0146 (Table 4). *L*. *sativa* at S_2 has the lowest DIM, whereas *B*. *vulgaris* at S_3 has the highest DIM, watered with T_3 and T_1, respectively (Table 5). The mean value of daily intake of iron was highest at T_2 S_2 at all T_1-treated sites (Fig 8).

The Fe health risk index varied from 0.002 to 0.022 (Table 4). *M*. *spicata* at S_2 had the lowest HRI, and *B*. *rapa* at S_1 had the highest HRI, both of which were irrigated with T_3 and T_1, respectively (Table 5). The mean value of the health risk index of iron was found to be maximum at S_1 and S_3 treated with T_1 (Fig 8).

Fe has a target hazard quotient of 0.055 to 0.47. THQ was lowest in *C*. *sativum* at S_2 and highest in *B*. *vulgaris* at S_3 watered with T_3 and T_1, respectively (Table 5). The mean value of the target hazard quotient was found to be maximum at S_3 treated with T_1 (Fig 8).

## Discussion

### Iron concentration in water

The iron concentration in all wastewater and groundwater samples in the present study was lower than the Fe concentrations (2.66 and 0.86 mg/L) in wastewater and groundwater samples, respectively, given by Alghobar and Suresha [43]. Sandeep, Vijayalatha [44] reported a much higher concentration of Fe in canal water and wastewater (40.90 and 238.59 mg/L, respectively) compared to the present findings. The iron level of all water samples was significantly lower than the allowed limit (5.0 mg/L) of WWF [45].

Iron was found in natural deposits, including corrosion of Fe-containing metals, refining of iron ores, industrial water, iron refining ores and wastewater [46]. Aquatic environments mostly face metal concentrations in higher amounts than permissible limits recommended for the safe use of animals, birds, fishes and humans [47].

### Soil iron

[48] reported a higher concentration of Fe in soil irrigated with wastewater (282.17 mgkg^-1^) than in the current investigated soil irrigated with wastewater. The Fe concentration in all sites and all treatments of groundwater and wastewater had higher Fe concentrations in the soil compared to the (1.21, 1.59 mgkg^-1^) findings of Fe in-ground and sewage water irrigated soils, as reported by [49]. Iron contents in the present investigation were found to be higher in wastewater- and canal water-treated soils at all sites than the maximum permissible limit (56.90 mgkg^-1^) given by Dosumu, Abdus-Salam [39]. Fe is an important nutrient of soil and is required for the proper growth of plants, but its higher concentration in soil may have toxic effects on the growth of plants [50].

Iron is a nutrient that plants require to survive. It accepts and donates electrons and is an essential component of photosynthesis and respiratory electron transport networks. However, when iron levels reach dangerously high levels, they become poisonous [51].

In the current research, high Fe contents were observed in soil samples irrigated with different sources of water containing higher iron than the maximum permissible value. To reduce the iron level in soil, the former can use fertilizers (NPK or NPK + lime) in a balanced manner. By using enough potassium (K) fertilizer with lime to acid soils and by removing organic matter (manure, straw) from soils with high Fe and organic matter content and decreasing wastewater irrigation [52].

### Iron concentration in crops

A higher level of Fe was reported by Chiroma, Ebewele [53] in food crops (883 mgkg^-1^) in Yola, (Nigeria) irrigated by urban wastewater, than the Fe value of the present findings in wastewater. The Fe concentration of crops (1.4 mgkg^-1^) in wastewater-cultivated food crops in the Agra Region in alluvial soil was reported by Parashar and Prasad [54] to be lower than that in the current work. The Fe concentration in crops grown on tube-well water sources had a higher value in the present investigation than (4.78 mgkg^-1^) reported by Yap, Adezrian [55]. According to the FAO/WHO [38] Iron levels in crops irrigated with a variety of treated water were considerably less than the permissible limit (425.5 mgkg^-1^). The continuous use of sewage water was the consequence of rising heavy metals in various parts of plants. Excessive use of pesticides and fertilizer, as well as their production from rocks in agricultural areas, were all probable causes of excessive levels of these metals [44].

Fe shortages in crops are entirely caused by excessive levels of calcium and bicarbonate [56]. High levels of calcium carbonate and pH can further limit iron availability to plants, and even a one-unit rise in pH can reduce Fe solubility by 1000-fold [57]. Since Fe was stationary in plants, the development of new leaves was the first sign of a deficit. Fe shortages also cause chlorosis in early leaf tissue [58]. Interveinal chlorosis was caused by a minor iron deficit that was sometimes mistaken for manganese reduction. Plants suffer from Fe shortages even though Fe is abundant in soil due to its poor solubility [59]. Fe shortages in crops might be caused by high pH, soil chemical characteristics, and insufficient solubility [60].

### Bioconcentration factor, pollution load index and enrichment factor of iron

BCF was found lower than the investigation of Khan, Ahmad [61] (0.4) in all types of samples irrigated with three different treatments of water at Different Sites. Hadif, Rahim [62] reported (0.00) BCF value lower than present findings. The results of the BCF data analysis showed that there was heterogeneity within treatments and among types. Plant metal uptake was affected by soil types, soil metal levels, soil pH, cation exchange capacity, and various crop types [63]. The BCF was lesser than one, the plants were not considered accumulators as suggested by [64]. for the present study for iron metal.

When compared to the results of Ahmad, Ashfaq [65] the iron PLI was found to be higher than (0.11and 0.12). Ololade [66] (1.06–1.1) and Izah, Bassey [67] (1.05–1.14) reported comparable. The PLI results for Fe in this study were lower than the Fe reference values (56.90) proposed by Dosumu, Abdus-Salam [39]. PLI values more than one indicate polluted soil, whereas values less than one indicate uncontaminated soil [68]. The PLI value for Fe in the current work was greater than unity, so the soil was contaminated with Fe.

Sarwar, Shahid [69] reported a higher value of EF for iron metal (386) in wastewater irrigated soil than present findings. Ahmad, Kokab [70] reported a higher value to present findings (0.134). Plants cannot take up iron because it forms chelates with the organic stuff in the clay, which prevents its absorption [71]. The enrichment factor had been used to understand the influence of natural and anthropogenic sources on heavy metals accumulation in soils. The enrichment factor was used to look at the impact of natural and human factors in soil heavy metal development [72].

### Daily intake, health risk, target hazard quotient of iron metal

Ahmad, Kokab [70] reported the daily consumption of Fe in-ground and wastewater grown samples (0.004, 0.02 mgkg^-1^day^-1^, respectively) that was similarly described in the present investigation. Khan, Ahmad [61] reported a higher value of Fe metal (0.04, 0.03 mgkg^-1^day^-1^) intake than present findings in different water treatments. The daily Fe consumption amount in all the samples was less than the daily tolerable limit (45 mgkg^-1^day^-1^) as suggested by USEPA (2002).

Khan, Iqbal [73] discovered a comparable range of HRI (0.01, 0.02) for Fe when irrigated with diverse sources of water, as had the current investigation. The health risk score for Fe in-ground and wastewater irrigated samples (0.06, 0.04) was found to be higher than the current values Khan, Ahmad [74]. Because Fe was such a vital element, it may be found in all living beings. It was believed to be on the border between macronutrients and micronutrients. In the current investigation, all the samples had HRI values less than one, indicating that there was no Fe toxicity in the food chain because of eating such crops. Because the Fe content does not exceed the maximum permitted and daily tolerated limits, the edible part of food crops can be consumed.

Balkhair and Ashraf [75] indicated a Target Hazard Quotient value for Fe (0.442) in crops cultivated in sewage water that was within range of the current findings. [Sanaei, Amin [76]] reported a THQ value for Fe in wastewater-irrigated crops of (0.001), which was lower than the current data. At all sites, the THQ for Fe was less than 1, indicating that Fe had no carcinogenic consequences on residents who consume these crops wastewater irrigated crops.

## Conclusion

The current study helps to offer an analysis of the impact of different soils located on different sites treated with T_1, T_2, and T_3 on Fe accumulation in selected food crops and estimates the potential health hazards for humans. The results revealed that wastewater-treated crops received the highest Fe uptake, but all of the observed Fe concentrations in the soil and food crop parts were lower than the FAO/WHO guidelines. The Fe content in edible portions was found to be safe for human consumption. The PLI values indicate pollution and poor soil quality due to the accumulation of Fe metal in the soil. The wastewater increases Fe buildup from the soil to the food crop system. Soil treated with wastewater elevates the organic matter content of the soil, which is a significant aspect of decreasing the Fe transfer from the soil to food crops and, subsequently, increasing iron in the food chain. However, the results show that the treatment of wastewater significantly contaminates food crops compared to canal water and tube well water, posing human health risks. As a result, municipal wastewater should be treated before being used for irrigation to avoid the detrimental effects of metal poisoning on humans. The government should cooperate with farmers to educate them and devise methods to protect public health.

## Supporting information

S1 Data (DOCX)
