# Expression of Concern: Health risk implications of iron in wastewater soil-food crops grown in the vicinity of peri urban areas of the District Sargodha

**DOI:** 10.1371/journal.pone.0358627

**Published:** 2026-09-21

**Authors:** The PLOS One Editors

The *PLOS One* Editors issue an Expression of Concern for this article [1] to notify readers of concerns about the article’s compliance with the PLOS Authorship policy.

Readers are advised to interpret the article [1] with caution.

Additionally, the following errors were noted in the published article and are corrected with this notice. These issues did not contribute to the decision to publish this Expression of Concern.

In the Materials and methods section, there is an error in the third sentence of the first paragraph. The correct sentence is: The soil and crop samples (leafy and root vegetables) were obtained from 3 different places in the district of Sargodha.

In the Iron concentration in water subsection of the Results section, there is an error in the fourth sentence of the first paragraph. The correct sentence is: At all sites, the descending order of iron metal in water was T_1 > T_2 and T_3 (Fig 1).

In the Iron concentration in soil subsection of the Results section, there are errors in the fifth and sixth sentences of the first paragraph. The correct sentences are: The descending order of Fe metal in soil at S_3) was reported to be *B. vulgaris > B. rapa > D. carota > S. oleracea > B. campestris > T. foenum-graecum > C. album > M. spicata > C. sativum* and *L. sativa* (Fig 2). At S_1, S_2, and S_3, the mean values of soil Fe were 81.26, 74.92, and 82.52, respectively (Fig 3).

In the Iron concentration in crops subsection of the Results section, there is an error in the fourth sentence of the first paragraph. The correct sentence is: At S_1, S_2, and S_3, the mean values of crop Fe were 12.3, 11.197, and 12.53, respectively (Fig 3). Meanwhile, the correct first sentence of its second paragraph is: The descending order of Fe metal in crops at S_1 was *B. rapa > B. vulgaris > D. carota > R. sativus > S. oleracea > T. foenum-graecum > C. album > B. campestris > C. sativum > M. spicata* and *L. sativa*; at S_2, it was *B. vulgaris > B. rapa > D. carota > R. sativus > B. campestris > S. oleracea > C. album > C. sativum > L. sativa > T. foenum-graecum* and *M. spicata*; and at S_3, it was *B. vulgaris > B. rapa > D. carota > S. oleracea > R. sativus > T. foenum-graecum > C. album > B. campestris > C. sativum > M. spicata* and *L. sativa* (Fig 4).

There are errors in the captions for Figs 1-4. Please see the complete, correct Figs 1-4 captions below.

**Fig 1:**
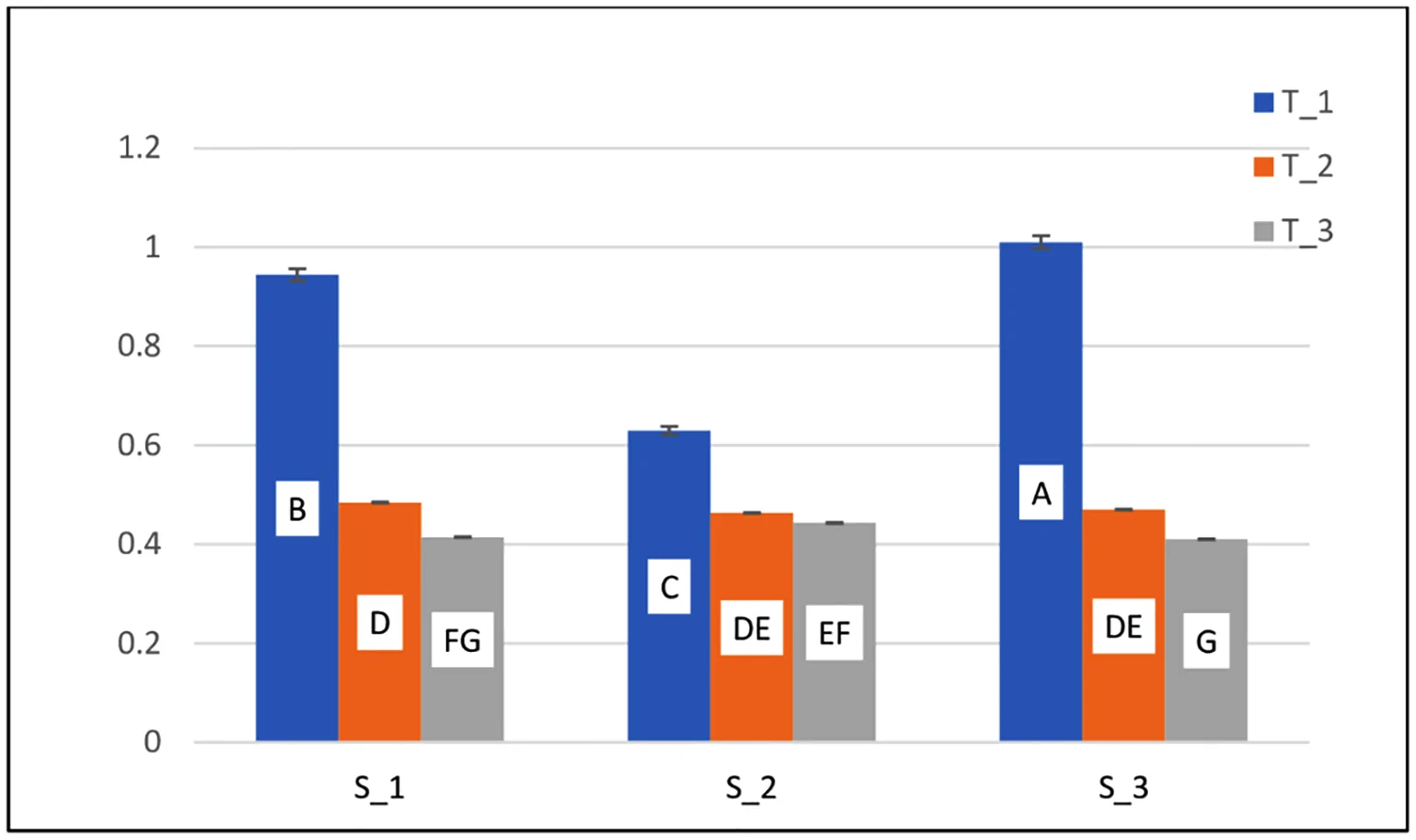
Fe (concentrations in mg/L) in water at three sites (S-1, S-2, and S-3).

**Fig 2:**
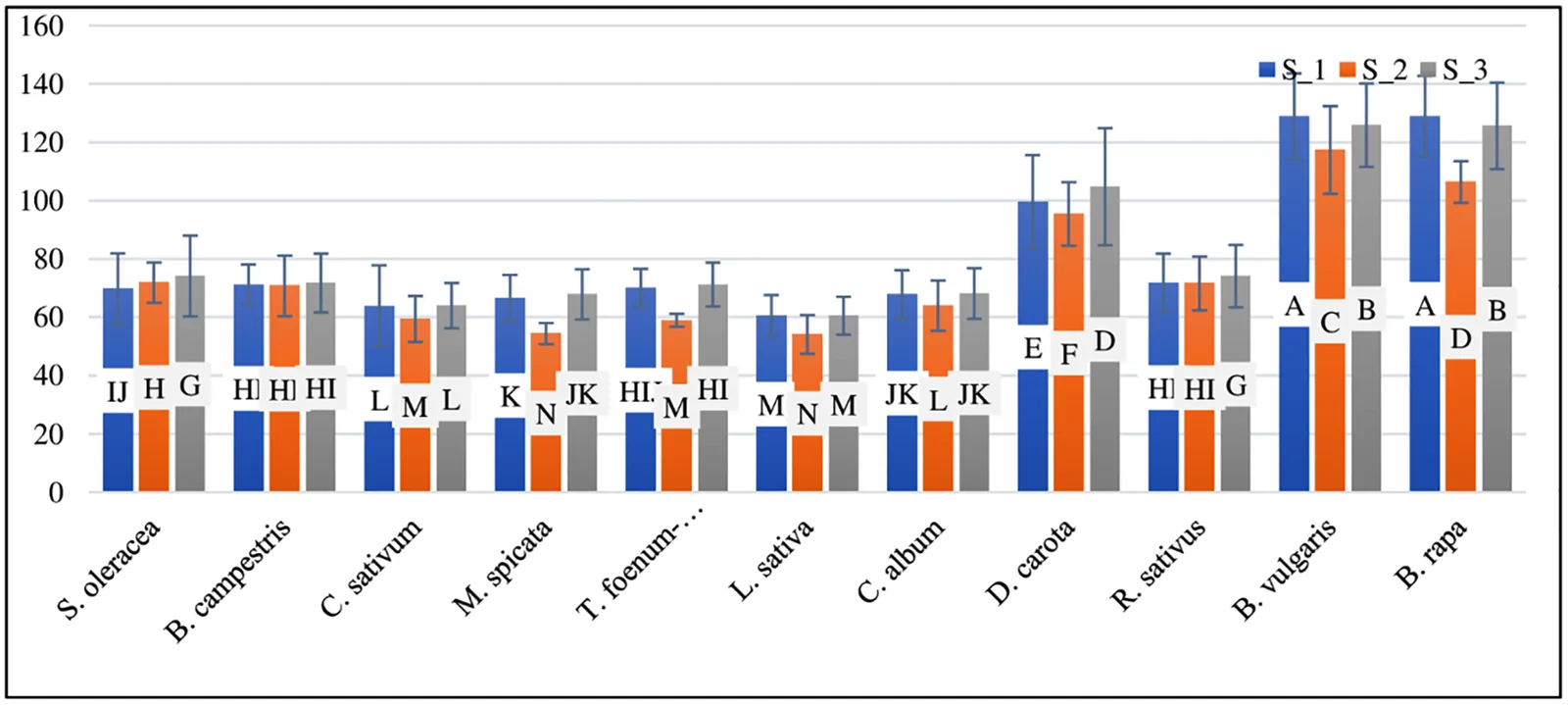
Fe (concentrations in mg/kg) in soil at S_1, S_2 & S_3.

**Fig 3:**
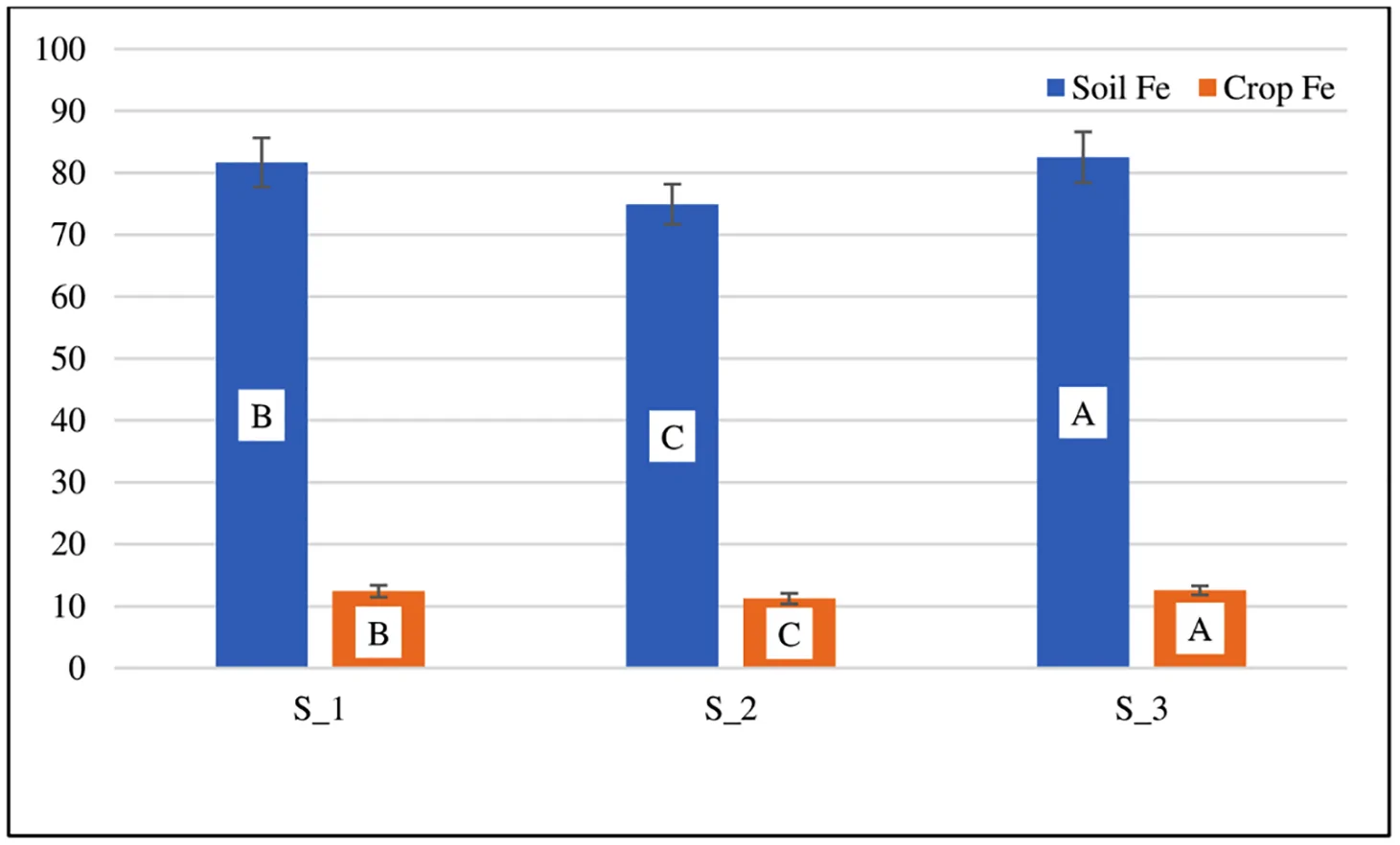
Fe (concentrations in mg/kg) at different sites in soils and crops.

**Fig 4:**
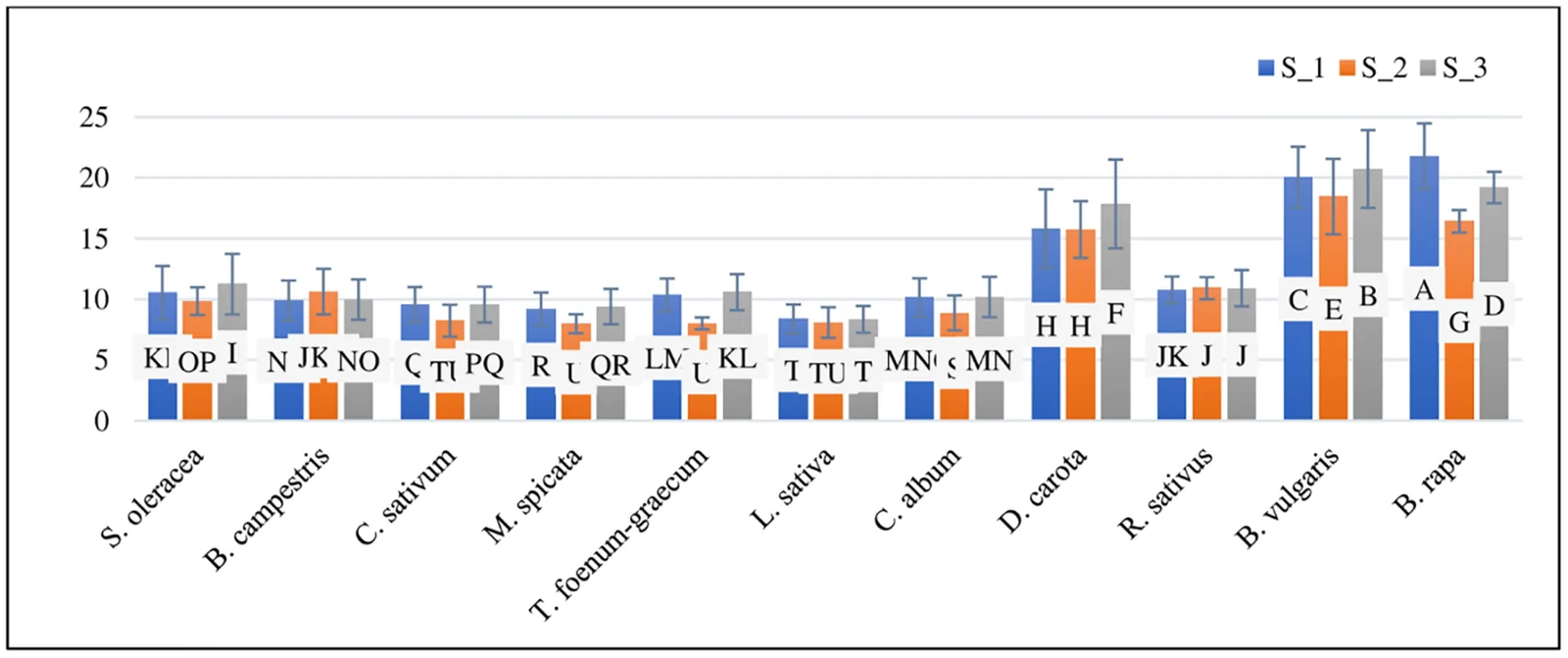
Fe (concentrations in mg/kg) in crops at three sites (S_1, S_2 & S_3).
