# Supplementary material for: Health risk implications of iron in wastewater soil-food crops grown in the vicinity of peri urban areas of the District Sargodha
Source: PLoS One. 2022 Nov 8;17(11):e0275497. doi: 10.1371/journal.pone.0275497 (PMC9642878; doi:10.1371/journal.pone.0275497)
Supplement: S1 Data — (DOCX) [file pone.0275497.s001.docx]

| General Linear Model: Fe versus site, Treatment | | | | |  |
| --- | --- | --- | --- | --- | --- |
| Analysis of Variance | | |  |  |  |
| Source | DF | Adj SS | Adj MS | F-Value | P-Value |
| Site | 2 | 0.0739 | 0.03695 | 281.62 | 0 |
| Treatment | 2 | 1.03878 | 0.51939 | 3958.85 | 0 |
| Site*Treatment | 4 | 0.17714 | 0.04428 | 337.54 | 0 |
| Error | 18 | 0.00236 | 0.00013 |  |  |
| Total | 26 | 1.29218 |  |  |  |
|  |  |  |  |  |  |
|  |  |  |  |  |  |
| Source | DF | Water |  |  |  |
| Site | 2 | 0.037* |  |  |  |
| Treatment | 2 | 0.519* |  |  |  |
| Site*Treatment | 4 | 0.044* |  |  |  |
| Error | 18 |  |  |  |  |
| Total | 26 |  |  |  |  |

| General Linear Model: Soil Fe versus Site, treatment, Crop soil | | | | | | | | | | | |
| --- | --- | --- | --- | --- | --- | --- | --- | --- | --- | --- | --- |
| Analysis of Variance | | | | | |  | |  | |  | |
| Source | DF | | | Adj SS | | Adj MS | | F-Value | | P-Value | |
| Site | 2 | | | 3444 | | 1722 | | 1259.64 | | 0 | |
| Treatment | 2 | | | 224264 | | 112132 | | 82023.7 | | 0 | |
| Soil | 10 | | | 150649 | | 15065 | | 11019.9 | | 0 | |
| Site x treatment | 4 | | | 7552 | | 1888 | | 1381.08 | | 0 | |
| Site x Crop | 20 | | | 2648 | | 132 | | 96.84 | | 0 | |
| Treatment*Crop soil | 20 | | | 23589 | | 1179 | | 862.77 | | 0 | |
| Site x Treatment x Soil | 40 | | | 9613 | | 240 | | 175.8 | | 0 | |
| Error | 198 | | | 271 | | 1 | |  | |  | |
| Total | 296 | | | 422030 | |  | |  | |  | |
| General Linear Model: Crop Fe versus Site, treatment, Crop soil | | | | | | | | | | |  |
| Analysis of Variance | | | | |  | |  | |  | |  |
| Source | | DF | Adj SS | | Adj MS | | F-Value | | P-Value | |  |
| Site | | 2 | 107.5 | | 53.77 | | 1794.73 | | 0 | |  |
| Treatment | | 2 | 6687 | | 3343.49 | | 111589 | | 0 | |  |
| Crop | | 10 | 4867.6 | | 486.76 | | 16245.7 | | 0 | |  |
| Site*treatment | | 4 | 240.4 | | 60.11 | | 2006.01 | | 0 | |  |
| Site*Crop | | 20 | 150.7 | | 7.54 | | 251.49 | | 0 | |  |
| Treatment*Crop soil | | 20 | 1069.9 | | 53.49 | | 1785.33 | | 0 | |  |
| Site*treatment*Crop soil | | 40 | 458 | | 11.45 | | 382.15 | | 0 | |  |
| Error | | 198 | 5.9 | | 0.03 | |  | |  | |  |
| Total | | 296 | 13587.1 | |  | |  | |  | |  |
| Source | | DF | Mean Squares (Soil) | | Source | | DF | | Mean Squares (Crop) | |  |
| Site | | 2 | 1722* | | Site | | 2 | | 53.77* | |  |
| Treatment | | 2 | 112132* | | Treatment | | 2 | | 3343.49* | |  |
| Soil | | 10 | 15065* | | Crop | | 10 | | 486.76* | |  |
| Site x Treatment | | 4 | 1888* | | Site x Treatment | | 4 | | 60.11* | |  |
| Site x Soil | | 20 | 132* | | Site x Crop | | 20 | | 7.54* | |  |
| Site x Treatment | | 20 | 1179* | | Site x Crop | | 20 | | 53.49* | |  |
| Site x Treatment x Soil | | 40 | 240* | | Site x Treatment x Crop | | 40 | | 11.45* | |  |
| Error | | 198 | 1 | | Error | | 198 | | 0.03 | |  |
| Total | | 296 |  | | Total | | 296 | |  | |  |

Soil graph

|  | S_1 | S_2 | S_3 |  | S_1 | S_2 | S_3 | S_1 | S_2 | S_3 |
| --- | --- | --- | --- | --- | --- | --- | --- | --- | --- | --- |
| *S. oleracea* | 69.706 | 71.855 | 74.131 |  | 12.2 | 6.91 | 13.9 | IJ | H | G |
| *B. campestris* | 71.206 | 70.752 | 71.72 |  | 6.91 | 10.4 | 10.1 | HI | HI | HI |
| *C. sativum* | 63.888 | 59.375 | 63.975 |  | 13.9 | 7.87 | 7.78 | L | M | L |
| *M. spicata* | 66.418 | 54.351 | 67.802 |  | 8.02 | 3.63 | 8.61 | K | N | JK |
| *T. foenum-graecum* | 69.924 | 58.941 | 71.234 |  | 6.62 | 2.2 | 7.5 | HIJ | M | HI |
| *L. sativa* | 60.523 | 54.136 | 60.486 |  | 7.05 | 6.64 | 6.5 | M | N | M |
| *C. album* | 67.898 | 63.94 | 68.119 |  | 8.23 | 8.59 | 8.7 | JK | L | JK |
| *D. carota* | 99.556 | 95.422 | 104.756 |  | 16.1 | 10.9 | 20.1 | E | F | D |
| *R. sativus* | 71.71 | 71.566 | 74.062 |  | 10.1 | 9.26 | 10.7 | HI | HI | G |
| *B. vulgaris* | 128.726 | 117.339 | 125.866 |  | 14.8 | 15 | 14.3 | A | C | B |
| *B. rapa* | 128.932 | 106.39 | 125.605 |  | 13.8 | 7.16 | 14.8 | A | D | B |

Crops graph

|  | S_1 | S_2 | S_3 |  | SE | SE | S_3 | S_1 | S_2 | S_3 |
| --- | --- | --- | --- | --- | --- | --- | --- | --- | --- | --- |
| *S. oleracea* | 10.5408 | 9.8498 | 11.2434 |  | 2.2 | 1.14 | 2.49 | KL | OP | I |
| *B. campestris* | 9.8856 | 10.6256 | 9.959 |  | 1.65 | 1.88 | 1.66 | NO | JKL | NO |
| *C. sativum* | 9.5348 | 8.2304 | 9.5497 |  | 1.47 | 1.31 | 1.48 | Q | TU | PQ |
| *M. spicata* | 9.1901 | 7.982 | 9.3939 |  | 1.36 | 0.776 | 1.45 | R | U | QR |
| *T. foenum-graecum* | 10.3504 | 8.0092 | 10.5794 |  | 1.35 | 0.491 | 1.49 | LM | U | KL |
| *L. sativa* | 8.3616 | 8.0806 | 8.3407 |  | 1.19 | 1.25 | 1.11 | T | TU | T |
| *C. album* | 10.1339 | 8.8699 | 10.1788 |  | 1.58 | 1.43 | 1.66 | MNO | S | MN |
| *D. carota* | 15.8086 | 15.7368 | 17.8492 |  | 3.23 | 2.34 | 3.66 | H | H | F |
| *R. sativus* | 10.7686 | 10.9101 | 10.9041 |  | 1.1 | 0.904 | 1.49 | JK | J | J |
| *B. vulgaris* | 20.0286 | 18.4522 | 20.7142 |  | 2.53 | 3.1 | 3.2 | C | E | B |
| *B. rapa* | 21.7641 | 16.418 | 19.2049 |  | 2.71 | 0.929 | 1.29 | A | G | D |

|  |  |  | indices graph | | |  |  |  |  |
| --- | --- | --- | --- | --- | --- | --- | --- | --- | --- |
|  | **indices mean value** | | | | | | | | |
|  | **S_1** | | | **S_2** | | | **S_3** | | |
|  | **T_1** | **T_2** | **T_3** | **T_1** | **T_2** | **T_3** | **T_1** | **T_2** | **T_3** |
| BCF | 0.16 | 0.14 | 0.14 | 0.15 | 0.14 | 0.14 | 0.16 | 0.14 | 0.14 |
| PLI | 2.14 | 1.25 | 0.92 | 1.8 | 1.6 | 0.84 | 2.21 | 1.23 | 0.91 |
| EF | 0.02 | 0.02 | 0.02 | 0.02 | 0.02 | 0.02 | 0.02 | 0.02 | 0.02 |
| DIM | 0.00879 | 0.00466 | 0.00333 | 0.00717 | 0.00606 | 0.00295 | 0.00898 | 0.0047 | 0.00329 |
| HRI | 0.013 | 0.007 | 0.005 | 0.01 | 0.009 | 0.004 | 0.013 | 0.007 | 0.005 |
| THQ | 0.281 | 0.148 | 0.105 | 0.228 | 0.195 | 0.093 | 0.287 | 0.15 | #NAME? |

|  | Stander Error | | | | | | | | |
| --- | --- | --- | --- | --- | --- | --- | --- | --- | --- |
|  | **S_1** | | | **S_2** | | | **S_3** | | |
|  | **T_1** | **T_2** | **T_3** | **T_1** | **T_2** | **T_3** | **T_1** | **T_2** | **T_3** |
| BCF | 0.003377123 | 0.005094 | 0.004435 | 0.004527 | 0.005238 | 0.004322 | 0.004491 | 0.004724 | 0.005793 |
| PLI | 0.195197294 | 0.122061 | 0.099573 | 0.164866 | 0.204643 | 0.078662 | 0.202094 | 0.119142 | 0.086387 |
| EF | 0.000536287 | 0.000724 | 0.000701 | 0.000678 | 0.000751 | 0.000674 | 0.00063 | 0.000715 | 0.000802 |
| DIM | 0.000967089 | 0.000547 | 0.000473 | 0.000794 | 0.000992 | 0.000353 | 0.000931 | 0.000628 | 0.000405 |
| HRI | 0.001441991 | 0.000821 | 0.000675 | 0.001129 | 0.001402 | 0.000486 | 0.001321 | 0.000867 | 0.000634 |
| THQ | 0.030494546 | 0.017258 | 0.015336 | 0.025578 | 0.031604 | 0.011447 | 0.029545 | 0.020045 | #NAME? |
